# Automatic Detection of Individual Trees from VHR Satellite Images Using Scale-Space Methods

**DOI:** 10.3390/s20247194

**Published:** 2020-12-15

**Authors:** Milad Mahour, Valentyn Tolpekin, Alfred Stein

**Affiliations:** 1 Faculty of Geo-Information Science and Earth Observation (ITC), University of Twente, 7514 AE Enschede, The Netherlands; milad.mahour@cgi.com (M.M.); valentyn.tolpekin@iceye.fi (V.T.); 2 CGI Nederland, 3068 AX Rotterdam, The Netherlands; 3 ICEYE, 02150 Espoo, Finland

**Keywords:** scale-space, discrete Gaussian, VHR, tree position, tree crown detection

## Abstract

This research investigates the use of scale-space theory to detect individual trees in orchards from very-high resolution (VHR) satellite images. Trees are characterized by blobs, for example, bell-shaped surfaces. Their modeling requires the identification of local maxima in Gaussian scale space, whereas location of the maxima in the scale direction provides information about the tree size. A two-step procedure relates the detected blobs to tree objects in the field. First, a Gaussian blob model identifies tree crowns in Gaussian scale space. Second, an improved tree crown model modifies this model in the scale direction. The procedures are tested on the following three representative cases: an area with vitellaria trees in Mali, an orchard with walnut trees in Iran, and one case with oil palm trees in Indonesia. The results show that the refined Gaussian blob model improves upon the traditional Gaussian blob model by effectively discriminating between false and correct detections and accurately identifying size and position of trees. A comparison with existing methods shows an improvement of 10–20% in true positive detections. We conclude that the presented two-step modeling procedure of tree crowns using Gaussian scale space is useful to automatically detect individual trees from VHR satellite images for at least three representative cases.

## 1. Introduction

Remote sensing (RS) imagery and technologies can help scientists to detect and delineate individual trees in precision orchard management. This is important information for efficient decision making regarding crop health and crop water requirement. Trees establish a spatial pattern at both coarse and fine spatial resolutions. These patterns can be characterized by different parameters such as shape, location, species, and crown size [1]. There is a need to precisely estimate tree parameters such as tree size and biomass from an image. In the past, the relatively coarse resolution of images was inadequate to delineate tree crowns individually [2]. Such images provided insufficient spectral and geometrical detail information. In contrast, high resolution images provide the necessary detailed information, for example, allows for identifying geometrical and spectral information of adjacent tree canopy interlocks.

**Figure 1:**
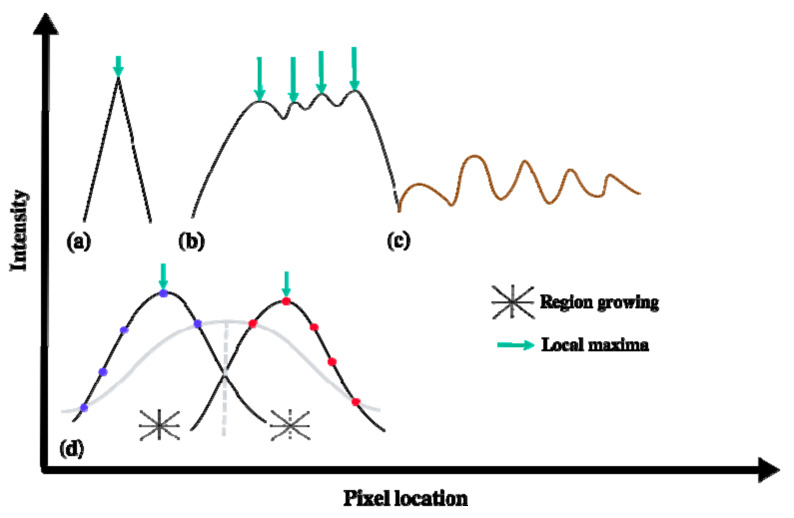
Intensity profiles. (**a**) A coniferous tree with a single maximum; (**b**) A broad-leaved tree with several local maxima; (**c**) A noisy background; (**d**) Represents the fitted Gaussian functions after region growing between two interlocked trees, colored blue and red. The grey curve is obtained if the Gaussian function is fitted to all points, whereas the black curves are obtained if region growing is applied on the two separate tree objects.

Obtaining detailed and reliable information on each individual tree from RS images requires many spectral and geometrical details. In particular, WorldView-2 imagery (WV-2) contains panchromatic and multispectral images with spatial resolution of 0.5 m (panchromatic band) and 2 m (visible and NIR bands), respectively. This resolution level is still too coarse for delineating interlocking tree crown boundaries, despite several image processing techniques that have been proposed to do so. For instance, region-based image segmentation [3] requires prior knowledge information on tree shape and size; image template matching [4,5] is useful for trees of the same height, size, and species; and watershed segmentation [6,7,8,9,10] suffers from over and under segmentation due to differences in tree heights and variation in the density of tree crowns [11]. In addition, other methods have not solved this problem. Valley following [12] explores local minima of neighboring tree crown boundaries in a way similar to edge detection [13,14,15,16,17]. The local maxima technique [18,19] searches local maxima corresponding to image brightness values at the center of the trees [15]. This technique, however, suffers from two limitations, i.e., it only detects the location of the tree and it suffers from multiple detections in irregular tree crown surfaces (Figure 1b), mainly due to the presence of multiple local maxima occurring at a single tree due to complexity in tree branches and the presence of wood or grass as background noise. To overcome the multiple local maxima problem, one can apply a fitted pre-smoothed Gaussian filter, but in the presence of adjacent tree interlocks this results in inaccurate tree sizes and imprecise locations. Figure 1 illustrates the local maxima of a coniferous tree in a natural forest area and a broad-leaved tree with several local maxima in an orchard area, where the number of local maxima depends upon spatial and spectral resolution of the sensor. The local maxima technique has been used in several studies to detect coniferous trees, but for broad-leaved trees it is inconvenient.

To overcome the multiple maxima problem of the broad-leaved trees, Ardila et al. [2] proposed a pre-smoothing Gaussian filter on a tree crown, followed by fitting a two-dimensional Gaussian function. This method separated multiple trees prior to fitting (Figure 1d) and caused inaccuracies in tree size estimates and tree positions for interlocked trees in the presence of background noise. The parameters of the pre-smoothing filter were adjusted empirically, and depended upon the spatial resolution of images, tree size and the degree of irregularity of tree crowns. 

Gaussian scale-space theory [13,20,21,22,23] has been proposed to bridge the gap between complex object patterns, detailed geographical coordinates, and sizes of objects. It is based upon detection of local maxima in the scale-space domain, detecting trees by providing their location and diameter [24]. It has as an assumption that a bell-shaped function similar to the Gaussian function can describe tree intensity in space. Recently, in the context of unmanned aerial vehicle (UAV) images, attention has been given to histogram of oriented gradient (HOG) features and support vector machine (SVM) classifiers [25], whereas a multiscale object-based approach has been used for monitoring the vegetation vigor in heterogeneous citrus and olive orchards [26]. 

The current paper follows up on Mahour et al. [24], where four limitations were identified. First, false detections occurred for trees that were cut in the past but were still identified as trees. Second, groups of trees were detected as a single tree. Third, tree size measurements were not consistently accurate. Fourth, the use of sampled Gaussian function in computation of scale-space methods failed to detect small trees resulted in false negatives. The present study aims at following up on that research by addressing the four limitations. Precise estimates for the parameters of a spatial tree intensity function are important to discriminate real trees from false detections, in particular if the contrast between trees and their background is low. Moreover, the presence of high frequency irregularities of tree crowns (Figure 1b) leads to problems in spatial modeling of the tree intensity function, and this can be compensated for by applying a smoothing filter. However, the parameters of the filter should depend upon tree size, image resolution, and the size of the irregularities such as width, height, and distance among them. In contrast, scale-space includes such filters implicitly, where small irregularities are only apparent at small scales, and such modeling remains unaffected at coarse scales. 

The objective of this research was to further improve automatic detection of individual trees from VHR satellite images. For this purpose, we model tree crown responses in the scale direction and introduce an improved real tree crown model by modifying a Gaussian blob model in Gaussian scale-space. The model is applied to the following three study areas: two orchards that are different in size, tree species, and planting pattern and one area with irregularly positioned trees.

## 2. Scale-Space Blob Detection

Scale-space theory provides a framework for objects that occur at different scales and positions and that can be observed from a two-dimensional grayscale image f(x,y). The Gaussian scale-space representation L(x,y;s). of f(x,y) is a family of derived smoothed images at different scales $\left(s\right)$ and locations (x,y) [27,28]:

(1) L(x,y;s)=g(x,y;s)∗f(x,y)

where ∗ denotes the convolution operator, and *s* ≥ 0. The function g(x,y;s) is either the two-dimensional sampled Gaussian kernel:

(2) $$g_{u}(x,y;s)=\frac{1}{2\pi s}e^{-\left(x^{2}+y^{2}\right)/2s}$$

or the two-dimensional discrete Gaussian kernel:

(3) $$g_{v}(x,y;s)=I_{x}(s)I_{y}(s)e^{-s}$$

In Model (3), $I_{n}(s)$, n=x,y, is the modified Bessel function of the first kind [29] with integer orders. The Gaussian smoothing kernel of Model (2), as the predominant part of the Gaussian scale-space, maintains the structure at a coarser scale similar to the finer scale of the original image. The initial condition of the Gaussian scale-space representation is that L(x,y;0) = f(x,y) at the finest scale, i.e., for s=0.

**Figure 2:**
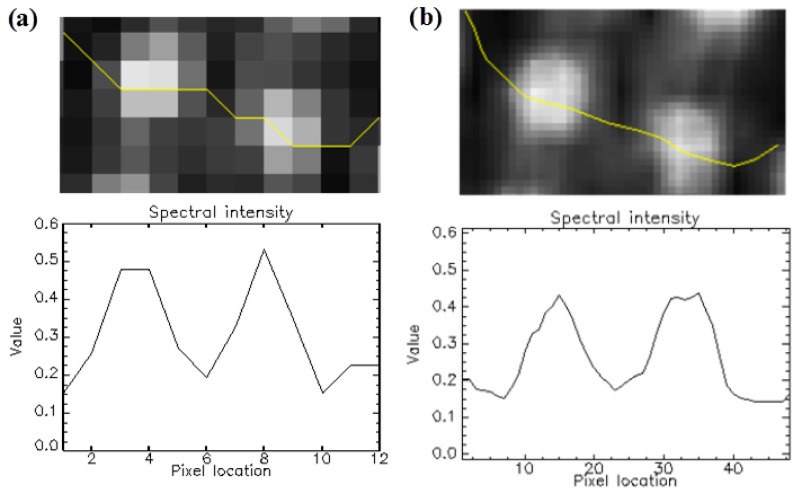
The spectral profile of tree crowns. (**a**) The NDVI image of WV-2 (2.0 m); (**b**) The NDVI image of WV-2 at 0.5 m spatial resolution.

A tree in a remote sensing image can be described by a bell-shaped spectral profile. The position of the brightest peak is subject to the position of the sun and the satellite sensor. A relative transformation, such as normalized difference vegetation index (NDVI), corrects for the uneven sun illumination of the crown [30]. Then, a tree object is a bright region on a dark background in an image with a smoothly varying intensity profile and a maximum at the center of the tree (Figure 2). We call such an object a blob that can be expressed as a function of geographical coordinates $\left(x_{0},y_{0}\right)$ and size of the tree $\left(r_{0}\right)$.

**Figure 3:**
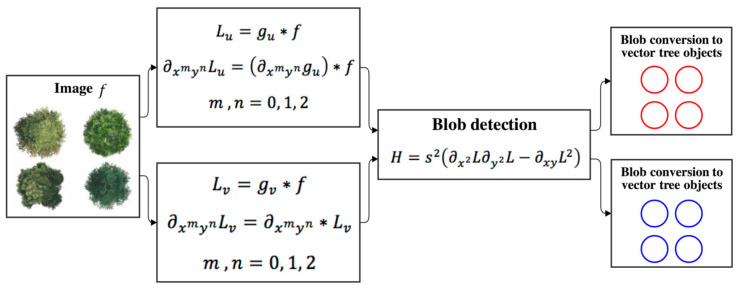
Schematic diagram of scale space for blob detection from either the two-dimensional sampled Gaussian kernel, indexed by u, or the two-dimensional discrete Gaussian kernel, indexed by v. The detected red and blue blob objects are from the sampled and discrete Gaussian kernels, respectively.

Figure 3 depicts how Gaussian scale-space theory is used for blob detection. Blobs are detected in scale-space using the local maxima of the scale-normalized determinant of the Hessian [31] as:

(4) $$H(x,y,s)=s^{2}\left(L_{xx}L_{yy}-L_{xy}^{2}\right)$$

where $L_{x}=\frac{\partial L}{\partial x}$. Due to the associativity of convolution, $L_{x}$ can be expressed as $\frac{\partial g_{u}}{\partial x}*f$, i.e., the original image f convolved by the kernel’s derivative $\frac{\partial g_{u}}{\partial x}$, performing a single raster convolution operation in sampled Gaussian scale space. In contrast, the computation of a derivative in discrete Gaussian scale space requires an additional convolution operation, $L_{x}=\partial_{x}*L_{v}$, where $\partial_{x}=\frac{1}{2}[-101]$.

We denote a local maximum of *H* over scale by its position $\left(x_{0},y_{0}\right)$, and size $s_{0}$. For a blob, $s_{0}=\frac{1}{2}r_{0}^{2}$. In the images, x,y and s have discrete values, and by interpolation the position $\left(x_{0},y_{0},s_{0}\right)$ of the local maximum is refined. 

## 3. Modeling Tree Crown in the Scale Direction

### 3.1. Gaussian Blob Models

For the Gaussian blob, the intensity profile is modeled as a Gaussian bell-shaped surface function centered at $x_{0}$ and $y_{0}$, and taking these values equal to 0, we obtain the following:

(5) $$G_{b}(x,y)=c+\frac{a}{2\pi s_{0}}e^{-\left(x^{2}+y^{2}\right)/2s_{0}}$$

where a describes the contrast between the blob and background, and the parameter c is the background assumed to be constant, i.e., independent from x and y, and $s_{0}>0$ specifies the size of the Gaussian blob. A scale-space extension of Model (5) includes the scale parameter s,

(6) L(x,y;s)=c+a2π(s0+s)e−(x2+y2)2(s0+s)

substituting Model (6) into Model (4) and setting x=y=0 and h(s)=H(0,0;s), we obtain a response of a Gaussian blob in the scale direction as:

(7) $$h(s)={\left(\frac{a}{2\pi }\right)}^{2}\cdot \frac{s^{2}}{{\left(s+s_{0}\right)}^{4}}$$

A major reason for considering the scale direction is that instead of the analysis of two-dimensional signal f(x,y), we analyze a one-dimensional signal h(s) and the noise associated with the background and irregularities of the tree crown is limited to small values of s. Model (7) is monotonically increasing for $0\leq s<s_{0}$ and decreasing for $s>s_{0}$. Its maximum occurs at $s=s_{0}$ and equals:

(8) $$h_{0}=h\left(s_{0}\right)={\left(\frac{a}{8\pi s_{0}}\right)}^{2}$$

A relatively simple model is the Gaussian blob model, where it is obtained from Model (7) as $f_{1}(s)=h(s)$. To account for the sub-pixel shift $\Delta r=\sqrt{\Delta x^{2}+{\Delta y}^{2}}$ between the Gaussian blob center and the discrete raster pixel location, we define $f_{2}(s)$ as an adjustment to $f_{1}(s)$ as:

(9) $$f_{2}(s)={\left(\frac{a}{2\pi }\right)}^{2}\cdot \left(\frac{s^{2}}{{\left(s+s_{0}\right)}^{2}}\cdot \frac{\left(s+s_{0}\right)-\Delta r^{2}}{{\left(s+s_{0}\right)}^{3}}\cdot e^{\frac{-\Delta r^{2}}{s+s_{0}}}\right)$$

The global maxima of model $f_{2}(s)$ with respect to s is attained at $s_{1}=s_{0}$ and $s_{2}=s_{0}+\frac{\Delta r^{2}}{2}$, assuming $\Delta r^{2}\ll s_{0}$.

### 3.2. Models for a Real Tree Crown

We observed in all our experiments that $f_{1}\left(s\right)$ and $f_{2}\left(s\right)$ are relatively crude approximations for real tree crowns especially for *s* > *s*_0_. To account for this, we modified the real tree model $f_{1}\left(s\right)$ as $f_{3}\left(s\right)$ by including a parameter δ>0:

(10) $$f_{3}(s)={\left(\frac{a}{2\pi }\right)}^{2}\cdot {\left(\frac{s}{{\left(s+s_{0}\right)}^{2}}\right)}^{2\delta }$$

This real tree model can again be shifted from the center by Δr, yielding model $f_{4}\left(s\right)$ as:

(11) $$f_{4}(s)={\left(\frac{a}{2\pi }\right)}^{2}\cdot {\left(\frac{s^{2}}{{\left(s+t_{0}\right)}^{2}}\cdot \frac{\left(s+s_{0}\right)-\Delta r^{2}}{{\left(s+s_{0}\right)}^{3}}\cdot e^{\frac{-\Delta r^{2}}{s+s_{0}}}\right)}^{\delta }$$

Upon implementation, the parameter *δ* is to be estimated, where *δ* ≠ 1 distinguishes *f*_3_(*s*) and *f*_4_(*s*) from *f*_1_(*s*) and *f*_2_(*s*), respectively. The global maxima of models *f*_3_(*s*) and *f*_4_(*s*) with respect to *s* are attained at *s = s*_3_ and *s = s*_4_, respectively. The physical interpretation of *δ* is that for increasing values of *δ* > 1 mainly the larger scales (*s* > *s*_0_) are affected. Therefore, introduction of *δ* leads to flexibility in the dependence modeling, where for larger values of *s* the dependence reduces. 

### 3.3. Implementation 

**Figure 4:**
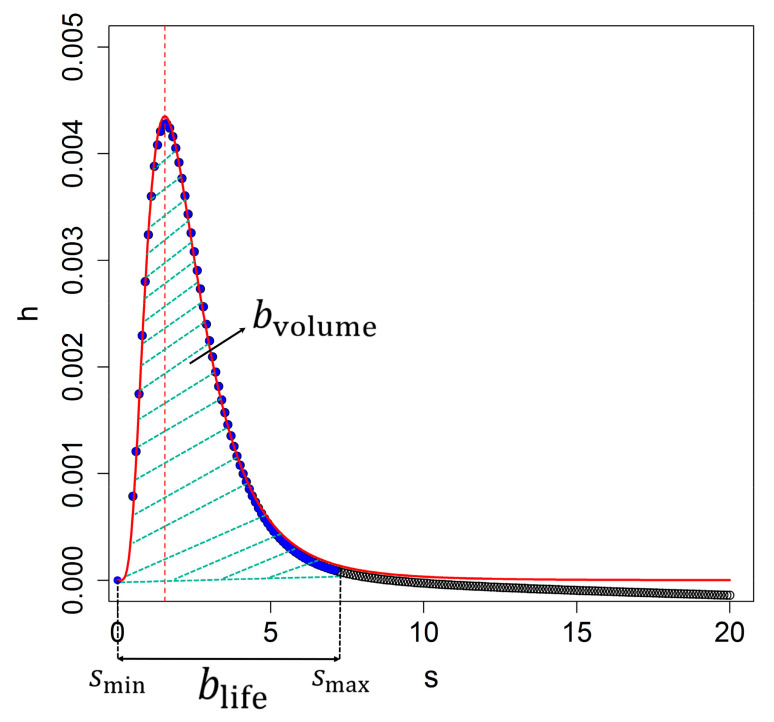
The blob volume *b*_volume_ and the blob lifetime *b*_life_. A blob volume is the area below the fitted model *f*_3_(*s*) at scale direction profiles *h*(*s*). The vertical dashed line indicates the location of the maximum *t*_0_ from model *f*_3_(*s*) (dashed line red color). Closed blue symbols show the range [*s*_min_, *s*_max_].

Upon implementation, we first identify the location of the local maximum with respect to space and scale on the discrete grid for x,y,s and refine it to $x_{0},y_{0},s_{0}$ using polynomial second order interpolation. Next, we identify the range $\left[s_{\min},s_{\max}\right]$ and $b_{life}=s_{\max}-s_{\min}$ corresponding to a single blob where $b_{life}$ is the lifetime. We do this by region growing into the direction *s* such that *h*(*s*) decreases if |*s* − *s*_0_| increases and *h*(*s*) exceeds a small positive threshold value (Figure 4). At *s > s*_0_, *h*(*s*) may include a contribution from the surrounding trees, for example, when several trees are found in a cluster. This contribution may affect fitting the models *f_k_*(*s*). Therefore, we limit the value to *s*_max_ = 2*s*_0_. Fitting all models to the discrete observations {*s_i_*,*h_i_*} in the range $\left[s_{\min},s_{\max}\right]$ was done by numerically minimizing the sum of the squares cost function using L-BFGS-B [32] and the tree size *r_k_* from all four models is determined from *s_k_* = $\frac{1}{2}$${r^{2}}_{k}$. The models (8)–(11) for *h* are proportional to *a*^2^, and therefore include dark objects (*a* < 0). We eliminate those by checking whether the scale-normalized Laplacian of the Gaussian scale space, i.e.,

(12) $$\Delta L(x,y,s)=s\left(L_{xx}+L_{yy}\right)$$

is at a minimum or at a maximum at *s*_0_. This is achieved by checking the sign of the second derivative of the ∆*L* with respect to *s*. The volume of a blob, *b*_volume_, is determined in the scale direction as:

(13) $$b_{\mathrm{volume}\;}=s_{\mathrm{life}\;}\int_{s_{\min}}^{s_{\max}}h(s)ds$$

being the area between *s*_min_ and *s*_max_ below *h*(*s*) (Figure 4). The blob volume is small for blobs with a low contrast with the background, whereas it is large for blobs with a high contrast. Relating blob volume to tree detection, we notice that a tree object has a larger scale-space volume, and hence the blob of a low volume can be discarded. In addition, the total relative error (*ε_r_*) for all models used to discriminate larger blobs consists of several tree objects and it equals:

(14) $$\varepsilon_{r}=\sqrt{\sum_{i=1}^{s_{\max}}{\left(\frac{f_{k}\left(s_{i}\right)-h\left(s_{i}\right)}{f\left(s_{0}\right)}\right)}^{2}}$$

for *k* = 1, …, 4. 

## 4. Uncertainty Assessment

Two uncertainty assessments were carried out to validate tree crown boundary detection, focusing on the presence and the spatial extent of individual trees. We follow [24] to accept the number of correctly detected tree objects, when the center of each falls within the spatial extent of the reference data for further analysis. Ref. [33] proposed over- and underestimation error metrics when assessing the area of a detected object $A_{D_{i}}$ with respect to the area of reference object $A_{R_{i}}$, as:

(15) $${\bar{A}}_{D_{i}}=1-\frac{\mathrm{area}\left(A_{D_{i}}\cap A_{R_{i}}\right)}{\mathrm{area}\left(A_{D_{i}}\right)}$$

and

(16) $$A_{D_{i}}=1-\frac{\mathrm{area}\left(A_{D_{i}}\cap A_{R_{i}}\right)}{\mathrm{area}\left(A_{R_{i}}\right)}$$

respectively, where *i* indexes individual objects. Models (15) and (16) both result in values between 0 and 1, and there is a good match between detected and reference tree crown boundaries $\left\{D_{i},R_{i}\right\}$ if these are close to zero. Furthermore, the total detection error is the following:

(17) $$\varepsilon_{D_{i}}=\sqrt{\frac{{\left({\bar{A}}_{D_{i}}\right)}^{2}+{\left(A_{D_{i}}\right)}^{2}}{2}}$$

with $0\leq \varepsilon_{D_{i}}\leq 1$. We consider three object accuracy indicators concerning the spatial extent of the tree objects, i.e., true positives (TP) where tree objects exist in the reference polygon layers, false positives (FP) that consider inappropriate detection where the trees are not present at the reference layer, and false negatives (FN) where there is a failure to detect objects in the reference layer [34]. The Euclidean distance (*E*) evaluates the distance between the centroid of a detected tree object $P=\left(x_{p},y_{p}\right)$ and the centroid of a reference tree crown boundary $O=\left(x_{o},y_{o}\right)$ as a positional accuracy indicator as:

(18) $$E=\sqrt{{\left(x_{o}-x_{p}\right)}^{2}+{\left(y_{o}-y_{p}\right)}^{2}}$$

We compared the scale space method with two other techniques for individual tree crown blob detections, i.e., the Laplacian of Gaussian (LoG) technique [21], and the Difference of Gaussian (DoG) technique [35]. LoG obtains the Laplacian of Gaussian images with successively increasing standard deviation, stacking them in a cube where blobs are identified as local maxima. Detection of larger blobs, however, is slower because of the larger kernel sizes during convolution. Additionally, only bright blobs, i.e., objects with higher NDVI values, on a dark background are detected. DoG detects blobs by finding maxima in the matrix of the determinants of the Hessian of an image. Bright blobs, i.e., with higher NDVI values on a dark background as well as dark blobs, i.e., with lower NDVI values are detected. However, DoG suffers from the same disadvantage as LoG for detecting larger blobs.

## 5. Study Area, Data and Tools

### 5.1. The Walnut Orchard

**Figure 5:**
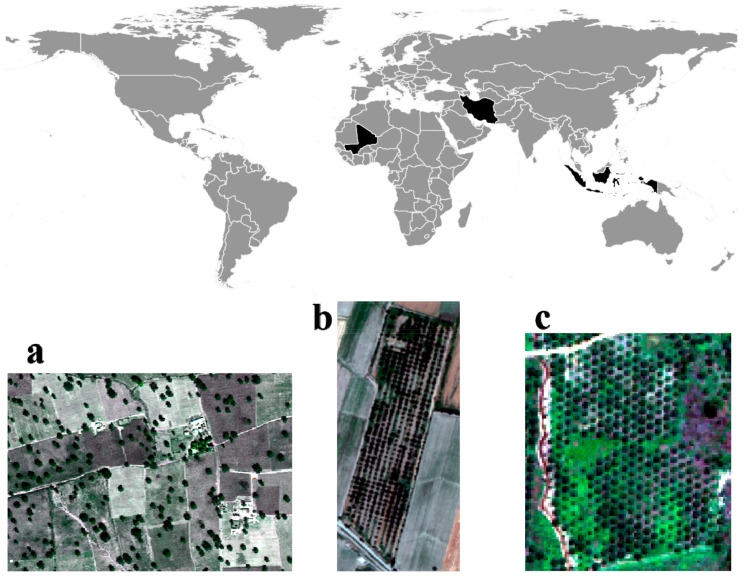
Three orchards at different locations displayed as true color composite from satellite images. (**a**) Vitellaria trees in Mali; (**b**) Walnut trees in Iran; (**c**) Oil palm trees in Indonesia.

The first study area is an orchard cultivated with walnut trees (Figure 5b) located in the Qazvin agricultural area in northwestern Iran with geographical coordinates 36°06ʹ N, 50°18ʹ E. The main reason for selecting this study site was the economic importance of the wood and nuts of the trees. The selected orchard contains trees of different crown shapes and sizes planted in rows. The study period was the middle of the growing season (July), when walnut tree crowns interlock with adjacent tree crowns. A four band (visible and near-infrared) multispectral image of WV-2 was taken on 7 July 2012, at a 2 m spatial resolution. An UltraCam digital image was collected on 17 July 2012 by a high resolution multichannel RGBI sensor, at a 0.5 m spatial resolution. The digital aerial image was delivered as an orthorectified image, corrected for lens distortion and camera tilt, and was co-registered to the panchromatic WV-2 image. The UltraCam image was used as a reference image for uncertainty assessment. For this validation, a polygon reference boundary layer was extracted manually from the UltraCam digital aerial photo using a visual interpretation.

### 5.2. Oil Palm Trees

The second study area is an orchard with oil palm trees (Figure 5c) planted in the Kutai Timur Regency, Kalimantan Timur province in Indonesia, located at geographical coordinates 00°58ʹ N, 118°03ʹ 0E. The oil palm trees are planted mainly with little differences in crown size and shape following a hexagonal pattern. The oil palm trees are commercially predominant in the food industry because of their fruit nutrition and oil production. A panchromatic image and a four band multispectral GeoEye image with visible and infrared bands were acquired on 25 May 2010, at a 0.5 m and a 2.0 m spatial resolution, respectively. The images were co-registered and projected in Universal Transfer Mercator (UTM) coordinates, with the standard spheroidal reference surface WGS84 and were geometrically corrected. The panchromatic GeoEye image was used as a reference image for validation. For this purpose, a polygon reference boundary layer was extracted manually representing the oil palm trees.

### 5.3. Vitellaria Trees

The third study area contains vitellaria trees (Figure 5a) located at 12°09ʹ N, 05°12ʹ 0W in Mali, commonly known as shea trees. This type of trees is found throughout Africa and their economic value is their oil rich seeds from which shea butter is extracted. In fact, the trees produce shea nuts for up to 200 years. A four band (visible and near-infrared) multispectral image of WV-2, and a panchromatic image were taken on 9 September 2015, at a 2 m and a 0.5 m spatial resolution, respectively. A polygon reference layer representing the vitellaria trees was manually extracted from the panchromatic WV-2 image.

### 5.4. Software

In this research, we used R software [36] with the Rcpp package [37] for Gaussian scale-space blob detection and tree crown modeling. Ggplot2 [38] was used for visualization and data analysis.

## 6. Results

### 6.1. Tree Detection in Discrete Gaussian Scale-Space

**Figure 6:**
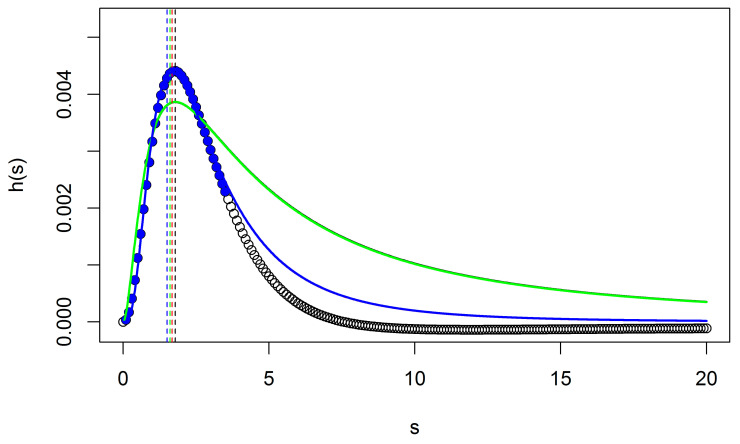
Scale direction profile *h*(*s*) for a real tree. The green line represents models *f*_1_(*s*), whereas the blue line represents *f*_3_(*s*). The red and black curve lines used for models *f*_4_(*s*) and *f*_2_(*s*) are invisible because their values are nearly identical to the blue and green curves, respectively. Green, black, blue, and red vertical dashed lines (left of the graph) indicate the location of the *s_k_* for the four models, whereas closed blue symbols show the obtained range [*s*_min_, *s*_max_].

Figure 6 shows the four applied models on the walnut trees detecting tree objects. From Figure 6, we observe that *f*_3_(*s*) and *f*_4_(*s*) provide a better fit to the data than *f*_1_(*s*) and *f*_1_(*s*). Next, we observe that the difference between *f*_3_(*s*) (*r* = 0.653) and *f*_4_(*s*) (*r* = 0.654) is negligible in terms of the parameter *s*_0_ that is related to tree size as compared with reference data. Moreover, the local maximum *s*_0_ of *f*_1_(*s*) (*r* = 0.578) and *f*_2_(*s*) (*r* = 0.579) are different from those of *f*_3_(*s*) and *f*_4_(*s*). For accurate estimation of *s*_0_, one needs either a large number of points at scale levels *s_i_* for local interpolation which is increasing computational time, or an accurate model, here *f*_4_(*s*). 

Table 1 gives the results in discrete Gaussian scale space using *f*_3_(*s*). It resulted in the detection of 391 individual trees out of 434 reference objects for the walnut orchard (90%), 252 individual trees out of 306 reference tree objects for oil palm trees (82%), and 238 trees out of 251 reference tree objects (94%) for vitellaria trees. FP error rates of 8.4%, 3.9%, and 5.0%, and FN error rates of 7.1%, 6.8% and 5.1% occur for walnut orchard, oil palm, and vitellaria trees, respectively. FN is lower for vitellaria trees because of the lower contrast between the trees and their background, whereas FP is lower for palm trees because of the low degree of interlocking adjacent tree crowns.

**Figure 7:**
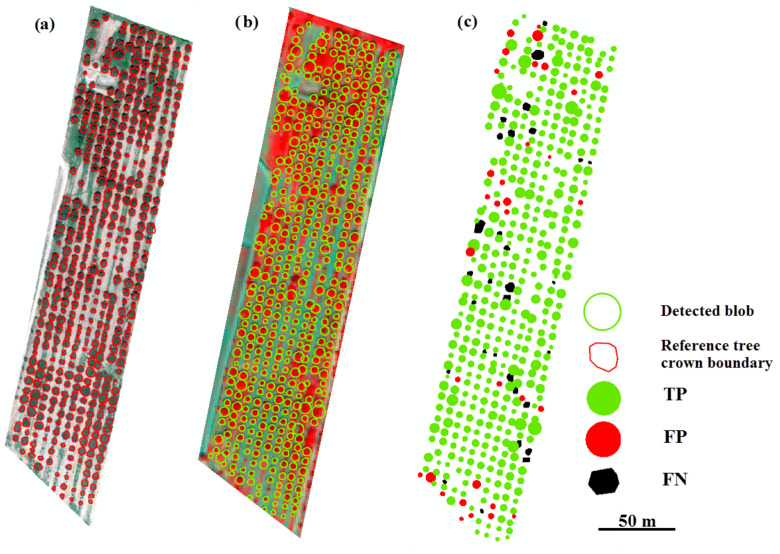
Results of tree detection in discrete Gaussian scale space with modified tree size measurement using model *f*_3_(*s*). (**a**) Reference data from an UltraCam image; (**b**) Walnut orchard with detected blobs; (**c**) The error map.

**Figure 8:**
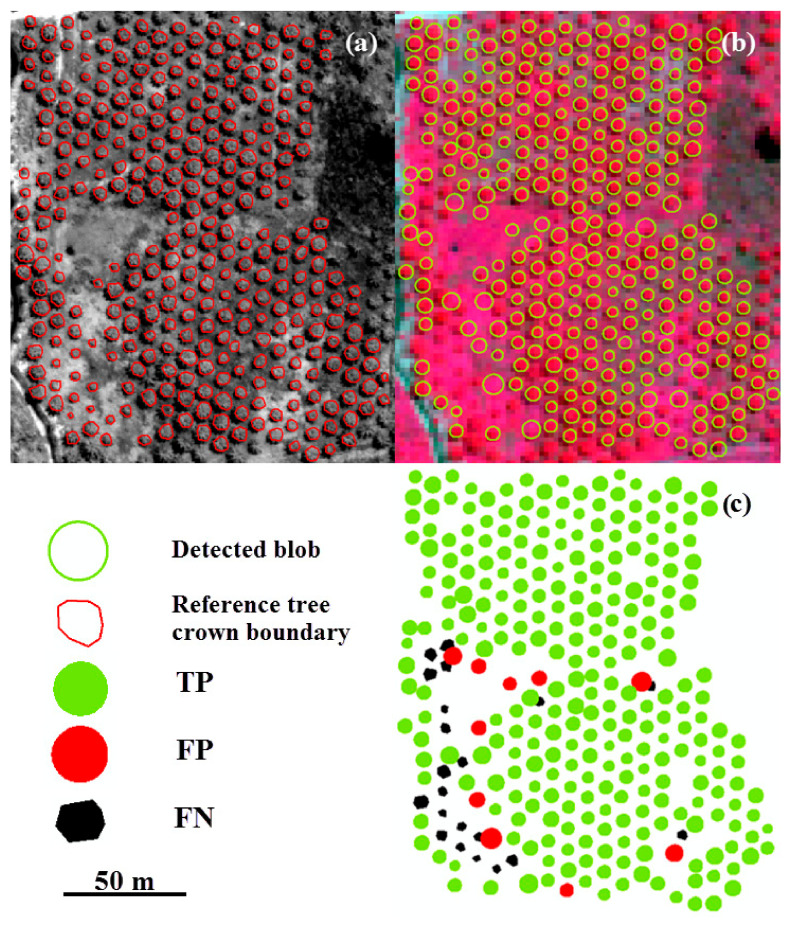
Results of tree detection in discrete Gaussian scale space with modified tree size measurements using *f*_3_(*s*). (**a**) Reference data from a panchromatic image; (**b**) The oil palm orchard with detected blobs; (**c**) The error map.

**Figure 9:**
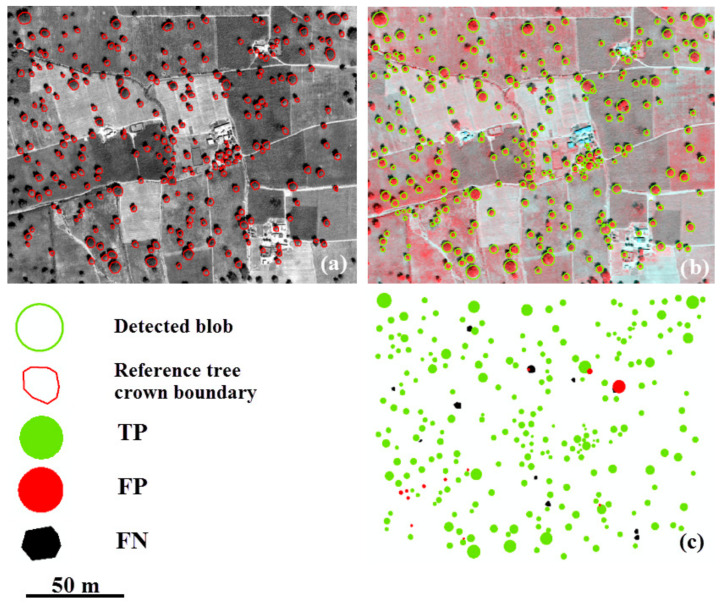
Results of tree detection in discrete Gaussian scale space with modified tree size measurements using *f*_3_(*s*). (**a**) Reference data from a panchromatic image; (**b**) Vitellaria trees with detected blobs; (**c**) The error map.

Figure 7, Figure 8 and Figure 9 illustrate the modeling of *f*_3_(*s*) for the different tree types, including a map of TP, FP, and FN. We observe that most trees have a different size parameter, ranging from 3.4 to 16.4 m for walnut, and from 1.5 to 19 m for vitellaria. Oil palm trees have similar range parameters, between 1.5 and 3 m. The high FP value for walnut (8.4%) is due to the regular tree pattern (Figure 7 and Figure 8). FNs for walnut and oil palm trees occur if adjacent tree crowns interlock, whereas, for vitellaria trees, they occur where the position of a detected blob centroid is outside the reference tree crown boundary.

Table 2 presents the positional inaccuracy of the different trees. For oil palm trees it is higher than for vitellaria trees, whereas for walnut trees the value of 0.91 m is slightly lower than that for vitellaria and oil palm trees. The reason is the different sun elevation angles during capturing of the VHR satellite images, resulting in small shadows, making identification of tree crown boundaries more difficult. Moreover, the aggregated ${\bar{A}}_{D_{i}}$ and ${\underline{A}}_{D_{i}}$ values for oil palm trees (0.42 and 0.33) are higher than those for walnut and vitellaria trees. The total detection error ($\varepsilon_{D_{i}}$) is lower for both walnut (0.27) and vitellaria (0.33) trees than for oil palms, because of their larger tree size and because they are located in an area with a clear contrast between background and trees.

### 6.2. Comparison between the Sampled Gaussian Kernel and the Discrete Gaussian Kernel

**Figure 10:**
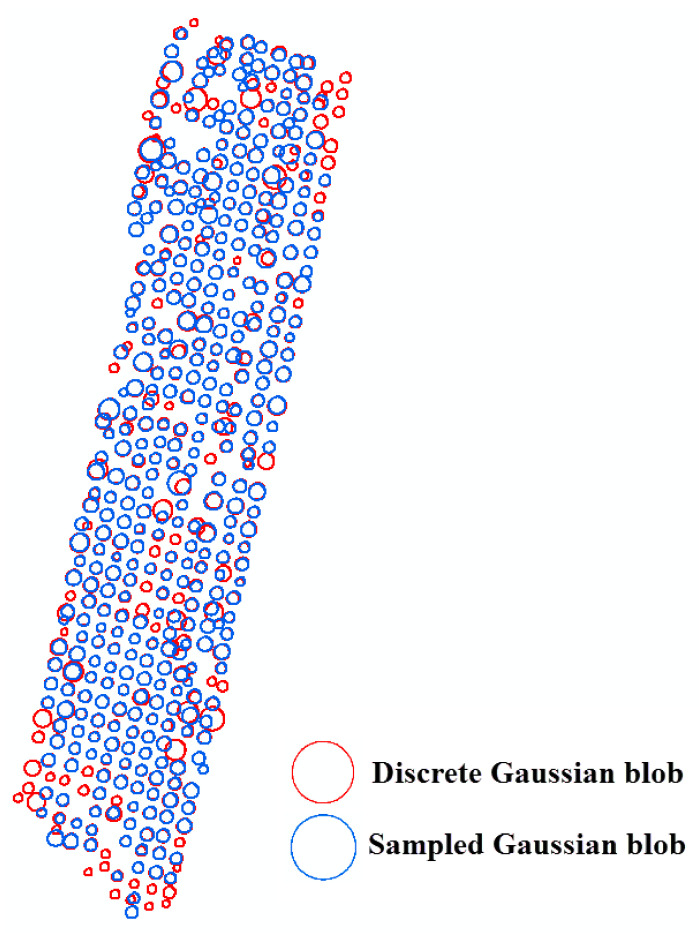
Differences between sampled and discrete Gaussian scale-space blob detection for the walnut orchard.

**Figure 11:**
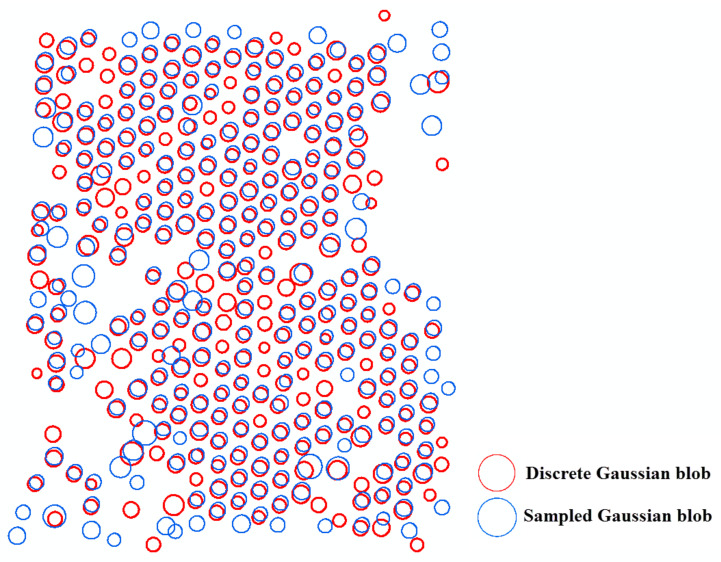
Differences between sampled and discrete Gaussian scale-space blob detection for the oil palm orchard.

**Figure 12:**
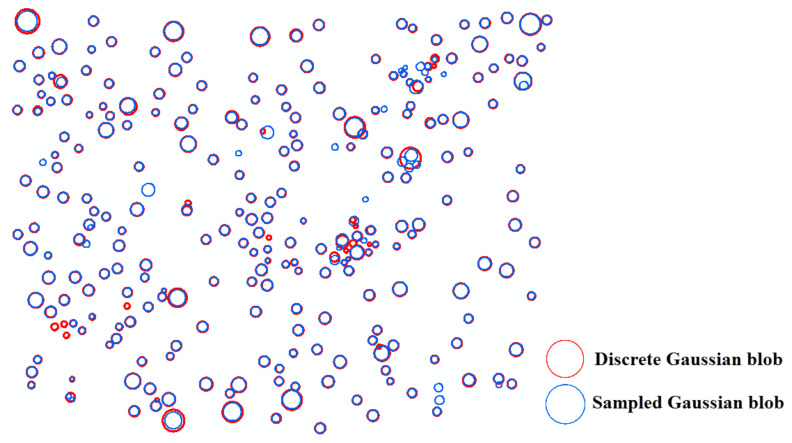
Differences between sampled and discrete Gaussian scale-space blob detection for vitellaria trees.

Figure 10, Figure 11 and Figure 12 compare the two-dimensional sampled Gaussian kernel *g_u_* and the two-dimensional discrete Gaussian kernel *g_v_* for walnut, oil palm, and vitellaria trees, respectively. Figure 10 shows that scale-space modeling with *g_v_* is capable of detecting 55 very small trees with a tree diameter lower than the pixel size as compared with the average size of walnut trees on the bottom and top rows of the orchard. The kernel *g_u_* performs better for the area where adjacent trees interlock. Figure 11 shows that, for oil palm trees, *g_v_* can detect 60 small trees more than *g_v_* in the hexagonal pattern, whereas *g_u_* performs better for detecting larger trees. For vitellaria trees, Figure 12 shows that *g_v_* detects 12 small trees, whereas *g_u_* is able to delineate trees with crown sizes that are larger than the pixel size. However, *g_v_* better approximates the local maxima at Model (4) when obtaining the derivatives at finer scales for small trees. 

### 6.3. Comparison among Tree Crown Modeling, LoG, and DoG

**Figure 13:**
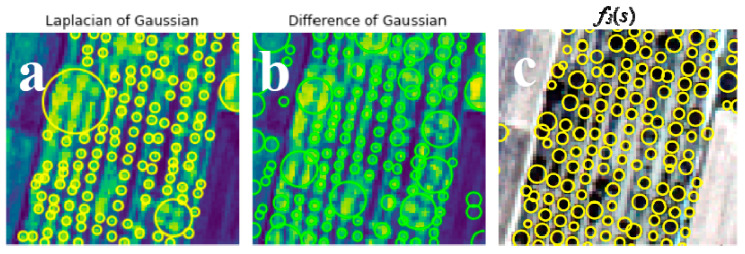
The Laplacian of Gaussian (LoG) technique (**a**), the Difference of Gaussian (DoG) technique (**b**), and (**c**) the discrete Gaussian scale space using *f*_3_(*s*) for the walnut tree orchard.

Comparing the discrete Gaussian scale space using *f*_3_(*s*) for the walnut tree orchard (Figure 13), Table 3 shows that it detects 391 individual trees from 434 reference objects. LoG detects 356 individual trees (82%), whereas DoG detects 311 trees (72%). FP error rates equal to 11.2% and 14.9%, and FN error rates of 11.9% and 14% occur for LoG and DoG, respectively. For the discrede Gaussian scale space using *f*_3_(*s*), both FP and FN are lower, whereas its TP is higher. The reason is that it more precisely detects complex tree crowns where adjacent tree crowns interlock.

### 6.4. The Parameter δ for Different Tree Types

**Figure 14:**
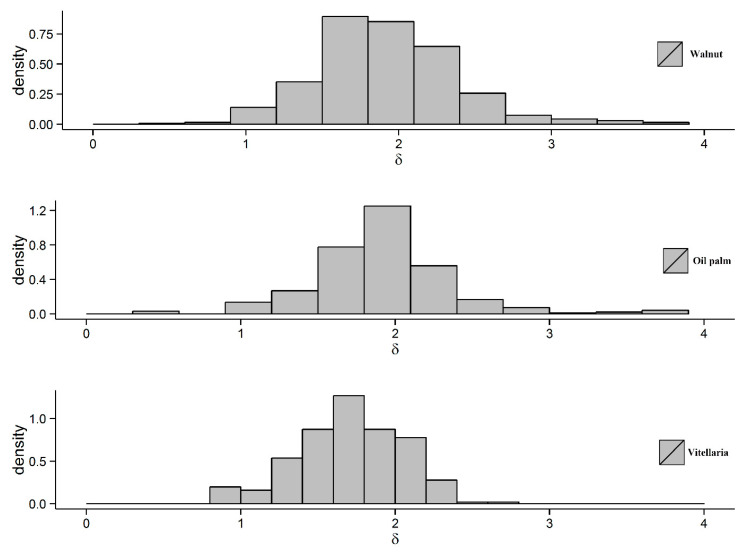
Distributions of parameter *δ* for different trees types obtained with model *f*_3_(*s*).

Figure 14 illustrates the density distributions of the parameter *δ* for different tree types, and for tree crown objects detected using *f*_3_(*s*) in discrete Gaussian scale space. For all the tree types, we observe a single mode for *δ* that is substantially larger than one. For vitellaria, the average *δ* is lower than for trees in the two types of orchards, whereas close values for *δ* are obtained for oil palm trees and for walnut trees. A likely explanation is that this is due to either tree species variation or irregularities in the spatial arrangement of the trees. 

The following three hypothesises relate to the parameter *δ*:

**Hypothesise 1(H_1_)**. *δ is related to the density of the trees, for example, the areas in an orchard, where trees are dense*.

**Hypothesise 2(H_2_)**. *δ is related to the contrast between a blob and the image background*.

**Hypothesise 3(H_3_)**. *δ is related to the tree size*.

As concerns H_1_, we considered the relation between *δ* and the density expressed as the number of trees per unit area (D_N_) and the density defined as expressed as area of the trees per unit area D_A_. Table 4 shows D_N_ and D_A_ values for the three trees orchards. The vitellaria orchard has lower D_N_ and D_A_ values as compared with the walnut and palm orchards, and also the average value of *δ* for vitellaria is lower (Figure 14). Moreover, the *δ* values for the palm and walnut orchards are similar. We observed the highest D_N_ value for the palm orchard, followed by the walnut and vitellaria orchards, whereas the D_A_ value is higher for the walnut orchard that contains larger trees of varying sizes, in contrast to the palm orchard that has trees of similar sizes.

As concerns H_2_, we observed that both walnut and palm orchards have a lower contrast between trees and background image as compared with vitellaria trees. Moreover, vitellaria trees are planted with a higher irregularity as compared to walnuts and palms. 

As concerns H_3_, we observed above that the parameter *δ* is insensitive of the left-hand side of *h*(*s*) (Figure 6).

## 7. Discussion

In this study, the intensity profile of a tree in the scale-space domain was characterized in an NDVI image by a bell-shaped surface as Gaussian blobs. The Gaussian blob models *f*_1_(*s*) and *f*_2_(*s*) inadequately described the trees, and their use caused overestimation and inaccurate tree size parameter estimation. The empirical models introduced in this paper, *f*_3_(*s*) and *f*_4_(*s*), provided a better description of the trees and more accurately estimated the tree size parameter *s*_0_. Indeed, models *f*_3_(*s*) and *f*_4_(*s*) adequately detected individual trees at the different scale levels (see Figure 6). All four models are asymmetric and in this sense their use extends local maximum interpolation as in [24]. Moreover, model *f*_3_(*s*) reduced the positional uncertainty of walnut trees from 103 cm in [24] to 91 cm in this study.

Models *f*_3_(*s*) and *f*_4_(*s*) provide a better fit to the data of Model (4) in the scale direction than models *f*_1_(*s*) and *f*_2_(*s*) because of the different *δ* values for individual trees. Differences between models *f*_3_(*s*) and *f*_4_(*s*) were negligible in this study, whereas the parameter Δr in *f*_4_(*s*) was affected by the resolution of the satellite image. For estimating trees under similar conditions as in this study, model *f*_3_(*s*) is to be preferred, as it is sparser and requires less input for estimating the tree crown parameters. In fact, model *f*_4_(*s*) more accurately estimates tree sizes as compared with model *f*_3_(*s*) with differences from 3 cm to 5 cm. This difference can be larger if coarse resolution pixels or smaller trees are considered.

Walnut and vitellaria trees both had a large variation in tree size. Figure 10 and Figure 12 show that in sampled Gaussian scale space we cannot detect trees with a slightly lower diameter than the pixel size of the image. The reason is that the local maxima of the blobs occur at finer scale levels than the image resolution. In contrast, in discrete Gaussian scale space, this problem is overcome, and we are able to also detect small trees at the finer scale level. In sampled Gaussian scale space, however, we are better able to delineate a large tree at coarser scale levels if adjacent trees interlock (Figure 10 and 11). 

The scale-space representation Model (1) is the convolution between the discrete original image *f* and the Gaussian kernel (*g_u_* or *g_v_*). For both Gaussian kernels, the convolution is associative. By taking the derivative of the sampled Gaussian kernel we can either convolve it with the original image $L_{x}=\partial_{x}L\;=\;\left(\partial_{x}g\right)*f$ or reconstruct a sampled image *f*, and then obtain the derivative $L_{x}=\partial_{x}L=\partial_{x}(g*f)$. As the derivative of the sampled Gaussian $\frac{\partial g_{u}}{\partial_{x}}\to \infty$ if s→0. As a result, the derivatives in scale space cannot be correctly computed for fine scales, corresponding to the smallest trees. Therefore, for small values of *s,* we used *g_v_* in Model (1) to effectively obtain and detect small trees.

Comparing the results of the discrete scale space with the well-known LoG and DoG techniques, we found that both LoG and DoG resulted in higher FP and FN. The reason is that the discrete scale-space technique performs better for individual tree crown detection where a symmetric pattern is observed with patches of trees. Modeling of tree crown in the scale direction of the determinant of Hessian, therefore, resulted in a superior technique as compared with LoG and DoG. 

We also compared the results of the presented scale-space methods with methods for tree detection in other studies. Brandberg and Walter [13] applied the multiple-scale algorithm to identify tree crown edge contours from local maxima on an aerial imagery at a 10 cm spatial resolution. For evaluation, they visually interpreted the mean area overlap between delineated and reference tree crown, resulting in an overall detection rate of 54%. In a forest area, Ref. [21] identified tree crown centers on two multispectral, pan-sharpened VHR satellite images, at a 60 cm spatial resolution. Using the estimated local maxima of the Laplacian in Gaussian scale-space on the classified RS imageries, they obtained detection rates of 93% and 86% for the first and the second images, respectively. Ref. [39] performed a plot-based method on the citrus orchard without tree crown interlock using aerial imagery, at a 50 cm spatial resolution. They combined image filtering with a weighted average filter and an unsupervised image classification followed by individualization of a tree using iterative local maximum filtering. They achieved an overall 90% detection rate, and a 40 cm positional error for individual trees. Ref. [40] used scale-space filtering on a papaya and a lemon orchard with isolated individual trees from images obtained by UAV. Applying the Laplacian of the Gaussian function resulted in a 95% detection rate for trees. FP (6%) and FN (5%) errors were due to multiple detections of a single tree, and even detection of a dead tree. 

All these studies were done on individual trees and delineation was done for different forest types and various tree species. In addition, different sensors and RS images were used with varying spatial and spectral resolution, making it difficult to compare the methods. Our method is able to automatically detect individual tree crowns at a slightly higher detection rate. In addition, the refined model *f*_3_(*s*) in the scale direction provides an accurate estimation of tree parameters for three economically important tree species.

A final issue we want to address concerns the precise boundary of the tree crown, which is undefined and has an unstable estimation. We note that tree crowns have fragmented boundaries, and that stability of its estimate, and hence of the size of a tree and its position, depend upon the wind speed, canopy density, and noise in the background. To estimate tree size, our method reports the scale parameter, *s*_0_, assuming that a circular shape gives the crown radius of an individual tree. Trees, however, may have an ellipsoidal or a truncated cone crown shape, or even have an asymmetric crown in other environments, such as in natural forests, where tree species and management practices, such as pruning, differ from those considered in this study. Therefore, other types of tree shapes should be considered in future modeling of tree crowns in scale space. Furthermore, a tree crown is partially transparent and it has fractal properties with a boundary depending upon spatial resolution of the observations. Hence, the precise boundary of a tree crown cannot be defined in a strict sense. Moreover, this boundary is not static due to wind induced motion of branches and leaves and pruning of tree crowns could affect the shape. 

A weakness of our method is that it is unclear whether it is able to address the presence of multiple maxima within a single tree. Multiple maxima can emerge due to several branches within a single tree. Trees may also be interlocking and form patches. This research can be extended by a more precise boundary estimation and extending the analysis towards identification of multiple maxima. 

Our study aimed at trees in orchards and isolated trees in small holder agricultural crop fields. Variation in spectral response of a single tree crown may exist, related mainly to the variation in leave density. Such variation has two main components as follows: (1) A low frequency radial dependence and (2) high frequency irregularities of tree crowns. The low frequency component can be observed from VHR satellite images of trees with a crown diameter of a few pixels. Therefore, a future focus could be to develop a mathematical model that describes the low frequency components. There are multiple needs for such a model which include the following: (1) It is able to detect trees and distinguish them from uneven noisy background. (2) It may help to differentiate different tree species. (3) It may help to investigate overall tree stress, related, for example, to health or water shortage. (4) It allows accurate position determination of individual trees. (5) It is more accurate than simple boundary delineation, because the boundary can be affected by wind, and by the different looking angles of sensor. A full development for such models is outside the scope of the current paper and should be addressed in future research.

This research considered two orchards and an area with isolated tree crowns of different shapes, sizes, and species. The scale of management is important in precision agriculture, as modern management of nutrient and water supply focuses on treatment of individual trees. An improved detection of individual trees and delineation of their crowns, as shown in this study, could likely improve irrigation management, which depends upon the type and crown size of a tree, being, in most cases, a function of the tree cover fraction [41]. 

## 8. Conclusions

In this research, we improved Gaussian scale-space modeling for detecting and delineating individual trees from VHR images on three tree types at different continents. We did so by discretizing a Gaussian blob model in the scale direction, thus, proposing a discrete Gaussian scale-space model. In doing so, we were able to more accurately determine the tree size and discriminate between real tree objects and false detections. The main finding from this study was that our proposed model provides a better description of real tree crowns than the Gaussian blob model. Our method reduced the number of false negatives, and the discrete Gaussian scale-space model resulted in better detection of small trees. It further showed a substantial improvement as compared with existing techniques. In this way, our work improved upon the automatic detection and delineation of trees with varying tree crown sizes, which was more accurate than existing models.

## Figures and Tables

**Table 1:** Detected tree objects based in discrete scale space, using model *f*_3_(*s*).

Tree Types	Reference	TP	FP	FN
Walnut	434	391 (90.09%)	33 (8.43%)	31 (7.14%)
Oil palm	306	252 (82.35%)	10 (3.96%)	21 (6.86%)
Vitellaria	251	238 (94.82%)	12 (5.04%)	13 (5.17%)

**Table 2:** Positional inaccuracy, overestimation, and underestimation metrics based upon the modified tree size measurement at *s*_0_ using model *f*_3_(*s*).

Tree Types	Positional [m]	${\bar{A}}_{D_{i}}$ [m]	${\underline{A}}_{D_{i}}$ [m]	$\varepsilon_{D_{i}}$ [m]
Walnut	0.91	0.32	0.13	0.27
Oil palm	1.50	0.42	0.33	0.57
Vitellaria	1.19	0.31	0.06	0.33

**Table 3:** Detected tree objects based in *f*_3_(*s*) model, Laplacian of Gaussian (LoG), and Difference of Gaussian (DoG) for walnut tree.

Models	Reference	TP	FP	FN
*f*_3_(*s*)	434	391 (90.09%)	33 (8.43%)	31 (7.14%)
LoG	434	356 (82.02%)	49 (11.29%)	52 (11.98%)
DoG	434	311 (71.65%)	65 (14.97%)	61 (14.05%)

**Table 4:** Densities expressed as the number of trees per unit area (D_N_) and as the area of trees per unit area (D_A_) for walnut, palm, and vitellaria trees.

Tree Type	D_N_	D_A_
Walnut	0.00819	0.252
Vitellaria	0.00019	0.017
Palm	0.01126	0.220
